# Another one bites the dust: faecal silica levels in large herbivores correlate with high-crowned teeth

**DOI:** 10.1098/rspb.2010.1939

**Published:** 2010-11-10

**Authors:** Jürgen Hummel, Eva Findeisen, Karl-Heinz Südekum, Irina Ruf, Thomas M. Kaiser, Martin Bucher, Marcus Clauss, Daryl Codron

**Affiliations:** 1 Institut für Tierwissenschaften, Universität Bonn, 53115 Bonn, Germany; 2 Steinmann-Institut für Geologie, Mineralogie und Paläontologie, Universität Bonn, 53115 Bonn, Germany; 3 Biozentrum Grindel und Zoologisches Museum, Universität Hamburg, Germany; 4 Zoo Zürich, Zürich, Switzerland; 5 Klinik für Zoo-, Heim-, und Wildtiere, Vetsuisse Faculty, Universität Zürich, Switzerland

**Keywords:** phytolith, grit, abrasiveness, hypsodonty

## Abstract

The circumstances of the evolution of hypsodonty (= high-crowned teeth) are a bone of contention. Hypsodonty is usually linked to diet abrasiveness, either from siliceous phytoliths (monocotyledons) or from grit (dusty environments). However, any empirical quantitative approach testing the relation of ingested silica and hypsodonty is lacking. In this study, faecal silica content was quantified as acid detergent insoluble ash and used as proxy for silica ingested by large African herbivores of different digestive types, feeding strategies and hypsodonty levels. Separate sample sets were used for the dry (*n* = 15 species) and wet (*n* = 13 species) season. Average faecal silica contents were 17–46 g kg^−1^ dry matter (DM) for browsing and 52–163 g kg^−1^ DM for grazing herbivores. No difference was detected between the wet (97.5 ± 14.4 g kg^−1^ DM) and dry season (93.5 ± 13.7 g kg^−1^ DM) faecal silica. In a phylogenetically controlled analysis, a strong positive correlation (dry season *r* = 0.80, *p* < 0.0005; wet season *r* = 0.74, *p* < 0.005) was found between hypsodonty index and faecal silica levels. While surprisingly our results do not indicate major seasonal changes in silica ingested, the correlation of faecal silica and hypsodonty supports a scenario of a dominant role of abrasive silica in the evolution of high-crowned teeth.

## 1. Introduction

Along with the spread of open landscapes and radiation of grasses during the Cenozoic (probably best documented for the Miocene), a striking morphological characteristic of dentitions evolved in different herbivore lineages [1–3]: hypsodonty, or high-crowned teeth. While the phenomenon apparently started to develop nearly 20 Ma, differences in crown height are also very obvious among extant grazers (hypsodont) and browsers (brachydont = low-crowned) [4–6].

It is generally agreed that the ultimate explanation for hypsodonty is the maintenance of functionality of teeth under conditions of increased wear [7]. The most accepted cause of increased wear is a rise of dietary silica content as a consequence of a higher proportion of grass in diets and/or foraging in open landscapes, respectively. Silica is harder than tooth enamel, and therefore critical for tooth wear [8]. There are several plant groups that are known for particularly high silica contents, like liver mosses or horsetails [9,10]. However, among angiosperms, grasses are best known to be silica accumulators, while dicots are generally characterized by lower silica contents. Surprisingly little data are available from direct comparisons, but the difference between grasses and browse (trees, shrubs, herbs) can generally be considered substantial: for example, in a study on East African vegetation, silica contents have been quantified to be 4.95 per cent dry matter (DM) in grasses compared with only 0.56–1.46% DM in browse [11] or in a sample of alpine plants to be 2.66 ± 1.60 (grasses) versus 0.20 ± 0.23% DM (dicots) [12]. C_4_ grasses generally seem to have higher values than C_3_ grasses [13,14].

Principally, dietary silica can occur as characteristic crystals in plant cell walls (phytoliths), or can be ingested as dust or contaminations of soil [5,15,16]. But while much of the discussion on the causes of hypsodonty focuses on whether phytoliths or grit should be considered the major abrasive agent (e.g. [17]), it should not be forgotten that even for a scenario disregarding this distinction and simply considering total silica, several inconsistencies and alternative explanations appear to exist: for example, if the rise of grasses is considered as the dominant trigger of hypsodonty, it is surprising that in the prime example of evolution of hypsodonty (Early to Middle Miocene of North America), the major rise of grasses appears to happen much earlier (4 Ma) than the onset of hypsodonty [2], described as ‘adaptive lag’ by Janis [3]. Increased tooth wear was also hypothesized to be caused not only by ingestion of abrasive silica, but also by higher general occlusal stress in combination with large quantities of low-quality food [18] or potentially also higher occlusal stress loads owing to a longer lifespan [19], the latter hypothesis being both rejected [18] and supported [20] later on. In addition, looking at the data of silica contents at the level of individual plant species, it appears that at least some dicots can reach fairly high silica levels [11], like Cucurbitaceae and Urticales [21] potentially rendering the ranking of grass and browse concerning their silica contents less unequivocal as often perceived. In fact, silica has been discussed as causing abrasion in dicot diets, too [22,23], and among hypsodont notoungulates, microwear indicated a browsing feeding style [24]. Once evolved, hypsodonty appears not to be decreased irrespective of a later shift to a less abrasive diet [18], which could imply a less tight connection of grass diets and hypsodonty and a generally high benefit/cost ratio of this dental characteristic.

Several studies have shown hypsodonty to be positively correlated to grass content of diet [5,25]. By contrast, it can be stated that while the focus of discussions is already on the distinction of the significance of different silica sources (exogenous dust versus endogenous plant phytoliths) for abrasiveness of herbivore diets, not even the relation of total ingested silica (sum of exogenous and endogenous silica) and hypsodonty has been tested yet in an empirical, quantitative assay.

A potential approach to tackle this data gap makes use of the fact that besides its mechanical resistance, a striking property of silica is its chemical stability and inertness. It is known to pass through the digestive tract without any significant degradation or absorption [26], characteristics qualifying silica as one of the standard markers in animal digestibility trials. This also opens the door for an estimate of tooth wear constraints faced by individual species owing to ingested silica: faecal silica should reflect ingested silica (as the sum of phytoliths and exogenous silica), integrating both diet (e.g. browse or grass) and habitat choice (e.g. open versus closed), and offering a way to approach the relation of ingested silica and hypsodonty.

Based on a sample of African herbivores, we tested how faecal silica levels reflect the degree of hypsodonty of a species, and to what extent faecal silica levels change between the wet and dry season.

## 2. Material and methods

Faecal samples were collected from 10 ruminants and five hindgut fermenters (table 1). In general, they were sampled for the dry and wet season at Kruger National Park, South Africa, except the two rhino taxa, which were both sampled at Lewa Wildlife Reserve, Kenya, and only for the dry season. All faeces were collected fresh shortly after observing defecation; care was taken not to contaminate samples with soil. After drying at 60°C they were milled through a 1 mm sieve. Silica content was quantified by using residual ash after boiling in acid detergent solution as used for acid detergent fibre (ADF) determination. All silica (biogenic and dust/soil) is recovered in this fraction (acid detergent insoluble ash—ADIA) [27], and according to Van Soest [28], the method is considered equivalent or even preferable to the classical method of acid insoluble ash (AIA) after Van Keulen & Young [29]. In the following, ADIA values are referred to as silica values if not explicitly indicated differently. The fibre bag system (Gerhardt, Königswinter, Germany) was used for sample analysis.

**Table 1.** Faecal silica contents of large African herbivores (mean ± s.d.; DM, dry matter).

	dry season			wet season		
	*n*	g kg^−1^ DM		*n*	g kg^−1^ DM	
greater kudu (*Tragelaphus scriptus*)	14	22	±6	20	26	±16
giraffe (*Giraffa camelopardalis*)	13	20	±6	18	24	±11
nyala (*Tragelaphus angasi*)	9	46	±12	6	30	±14
impala (*Aepyceros melampus*)	15	99	±28	20	147	±70
waterbuck (*Kobus ellipsiprymnus*)	5	117	±31	19	117	±32
sable antelope (*Hippotragus niger*)	8	59	±6	9	52	±10
roan antelope (*Hippotragus equinus*)	5	128	±10	7	95	±29
blue wildebeest (*Connochaetes taurinus*)	15	138	±11	19	132	±18
tsessebe (*Damaliscus lunatus*)	3	140	±28	16	131	±23
African buffalo (*Synceros caffer*)	15	146	±24	20	121	±20
black rhino (*Diceros bicornis*)	10	17	±6	—	—	
African elephant (*Loxodonta africana*)	20	47	±7	20	59	±14
warthog (*Phacochoerus aethiopicus*)	6	100	±12	6	163	±57
plains zebra (*Equus burchelli*)	6	126	±19	6	126	±16
white rhino (*Ceratotherium simum*)	10	75	±13	—	—	

Hypsodonty indices of the respective species were taken from the literature (primarily [5]; if the data of Mendoza & Palmqvist [4] differed, the average of both studies was used). Dietary information for each species was derived from stable carbon isotope analysis of faeces [30,31]. *δ*^13^C data from faeces were converted to estimates of the ratio of C_3_ browse to C_4_ grass in the diet of each sample using a simple linear mixing model that controls for spatio-temporal variations in the isotope composition of dietary baselines (plants) (see [31] and references therein).

We tested the hypothesis that hypsodonty is reflected in the silica content of faeces by correlating the hypsodonty index with the mean silica content of each species. In the same way, we tested the hypotheses that faecal silica content reflects proportions of browse and grass in the diet (estimated %C_4_ grass intake with the mean faecal silica content of each species), and that hypsodonty reflects proportions of browse and grass intake. *δ*^13^C data were bimodally distributed, and we thus used Spearman's rank correlations for the analyses. We controlled for phylogenetic effects in the analyses by linear regression through the origin of the independent contrasts of these same variables. The phylogenetic tree was based on the phylogeny proposed by Bininda-Emonds *et al*. [32], and branch lengths transformed by Pagel's (1992) method (dry season data) or Grafen's *ρ* (wet season data). Raw data were analysed with Statistica v. 8.0 [33], and independent contrasts analysis with the PDAP module for Mesquite v. 2.5 [34,35]. In all tests, dry and wet season data were analysed separately. For the comparison of wet and dry season data, the non-parametric Wilcoxon test for matched pairs was used.

## 3. Results

Faecal silica values ranged between 20 and 146 g kg^−1^ DM in ruminants and between 17 and 163 g kg^−1^ DM in hindgut fermenters (table 1). Values for browsers (17–46 g kg^−1^ DM) were lower than those of grazers (52–163 g kg^−1^ DM), with non-overlapping ranges. There was no overall difference in faecal silica contents between the dry and wet season (dry season: 93.5 ± 13.7 g kg^−1^ DM; wet season 97.5 ± 14.4 g kg^−1^ DM; *p* = 0.639) for all species, and also the exclusion of high-browsing and intermediate feeding species resulted in no significant difference (dry season: 111 ± 36.0 g kg^−1^ DM; wet season 107 ± 42.0 g kg^−1^ DM; *p* = 0.297).

**Figure 1.**
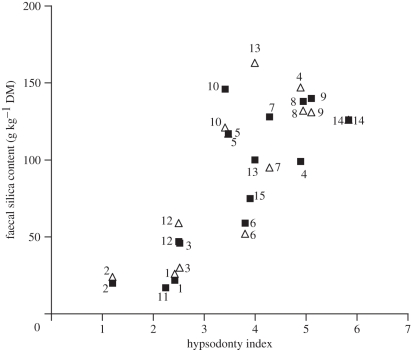
Correlation of faecal silica level and hypsodonty index [5] in large African herbivores (dry season: *n* = 15, *r* = 0.80, *p* < 0.0005; wet season: *n* = 13, *r* = 0.74, *p* < 0.005; phylogenetically controlled analysis 1, greater kudu; 2, giraffe; 3, nyala; 4, impala; 5, waterbuck; 6, sable antelope; 7, roan antelope; 8, blue wildebeest; 9, tsessebe; 10, African buffalo; 11, black rhino; 12, African elephant; 13, warthog; 14, plains zebra; 15, white rhino). Filled squares, dry season; open triangles, wet season.

As predicted, hypsodonty increased across species with increasing C_4_ intake, and with increasing faecal silica content (figure 1). These relationships were consistently significant in both seasons (although slightly more pronounced in the dry season), and were evident in raw data and the independent contrasts (table 2). For the phylogenetically controlled analysis, faecal silica content also was positively correlated to C_4_ grass in the diet in both dry and wet season data (table 2).

**Table 2.** Correlation analyses of relationships between faecal silica content and hypsodonty (hypsodonty index HI) and %C_4_ grass in the diet and between %C_4_ grass in diet and hypsodonty. *r*_s_, Spearman's correlation coefficient; *r*_p_, Pearson's product-moment correlation coefficient; %C_4_ in diet are data derived from *δ*^13^C of faeces [30,31]; HI, hypsodonty index ([5], combined with [4]).

variables	season	analysis of raw data			independent contrasts analysis		
		*n*	*r*_s_	*p*	d.f.	*r*_p_	*p*
faecal silica, HI	dry	15	0.76	0.0011	11	0.80	0.0004
	wet	13	0.77	0.0019	9	0.74	0.0037
%C_4_ in diet, faecal silica	dry	15	0.73	0.0019	11	0.79	0.0005
	wet	13	0.42	0.1557	9	0.76	0.0028
%C_4_ in diet, HI	dry	15	0.81	0.0002	11	0.75	0.0012
	wet	13	0.68	0.0103	9	0.76	0.0024

## 4. Discussion

Dietary silica is considered to exhibit negative effects on herbivores [36]; the mechanisms are discussed to work on several levels like diet digestibility [37,38], diet preference [39,40], bite rate [40] and even the development of pathological conditions like urolithiasis [41]; however, the negative effect of ingested silica (phytoliths plus dust and grit) is most renowned for a corresponding increase in diet abrasiveness (e.g. [5,8,42]). It is generally believed that tooth wear and dental abnormalities are important factors limiting the lifespan, the reproductive success and the body condition of free-ranging wild animals [43], although actual studies documenting this are still limited in number [20,44–50]. The effect of grit on tooth wear *in vivo* has, so far, only been investigated once in several populations of Australian sheep, in which tooth wear on incisors was a direct function of the amount of soil ingested [51], while laboratory approaches also found evidence for the abrasive effect of plants rich in phytoliths [39,52].

### (a) Correlation between faecal silica content and hypsodonty

Browsing or grazing feeding style was well reflected by faecal silica content in this study, on an overall species basis as well as when comparing browsing and grazing rhinos or ruminants like African buffalo and giraffe. The major goal of this study was to quantitatively approach the hypothesis of a direct correspondence between silica content (= abrasiveness) of the ingested material and the incidence of hypsodonty. We can state that the relation of hypsodonty and silica content was almost more obvious than we anticipated. The significant positive correlation between these traits was true for both seasons, and these results imply a considerable influence of ingested silica on hypsodonty.

A limited number of studies have reported faecal silica values of wild herbivores (table 3), and a small dataset of five North American ruminants [53] can be used as a control of the results of our study: In fact, these data are in accordance with our results since the hypsodonty index ranking of the five ruminant taxa is identical with that of faecal silica.

**Table 3.** Faecal silica contents reported in literature; hypsodonty index (HI) according to Janis [5] (DM, dry matter; AIA, acid-insoluble ash).

	silica content (% DM)	HI	method	reference
bighorn sheep (area 1)	May–July: 20–30, rest of year: <4	4.11	AIA	[56]
bighorn sheep (area 2)	May–July: ∼7–10, rest of year: negligible	4.11	AIA	
cattle	June: 12.8		AIA	[57]
	August: 18.0		AIA	
wildebeest	20.2	4.94	^a^	[36]
sheep				
high wear	24 (10–60)		AIA	[51]
medium wear	13 (2–35)		AIA	
low wear	9 (5–14)		AIA	
white-tailed deer	2.7	1.23	AIA	[53]
moose	5.4	1.34	AIA	
mule deer	6.5	1.59	AIA	
elk	7.1	1.96	AIA	
bison	15	4.87	AIA	

^a^According to Jones & Milne [58].

Obviously, we have to acknowledge that our data cannot totally exclude a contribution of other particularities of grasses (like higher occlusal forces) to the development of high-crowned teeth; however, our preferred and most likely interpretation is that of a causal relation of ingested silica levels, abrasiveness of ingested material and hypsodonty.

### (b) Influence of diet digestibility

When using faecal silica as a proxy for ingested silica, DM digestibility of the ingested diets could potentially interfere with faecal silica as a direct indicator of ingested silica, via different ‘dilution’ levels by indigestible material. This would translate in an overestimation of silica in more digestible, and the opposite in less digestible samples. When a lower DM digestibility of average browse compared with grass is assumed [54], e.g. 45 per cent for browse and 60 per cent for grass, correcting the average ruminant browser (28 g kg^−1^ DM) and grazer (115 g kg^−1^ DM) faecal silica value mathematically to an intermediate digestibility level results in values of 32 g kg^−1^ DM (browser) versus 97 g kg^−1^ DM (grazer), and even when assuming the most extreme imaginable difference in DM digestibility (40% for browsers versus 70% for grazers), a correction still results in values of 37 g kg^−1^ versus 77 g kg^−1^ DM of faecal silica in browsers versus grazers. While any interpretation of the values should keep in mind that it is concentrations and not amounts that are actually measured, it can be safely concluded that differences of the magnitude measured here will hold true irrespective of any realistic difference in digestibility.

### (c) Seasonal differences

Two major effects may influence ingested silica amounts in the dry season: first, the amount of browse in the diets of opportunistic feeders will increase, particularly in diets of mixed feeders, which should lead to an overall decrease in faecal silica in these taxa. Second, the amount of exogenous grit/dust is intuitively assumed to increase, leading to a general increase in faecal silica levels. In a study on the influence of overall rainfall on abrasion, using mesowear as a measure (the latter resulting from the combination of wear owing to abrasion = tooth–food contacts and attrition = tooth–tooth contacts), Kaiser & Rössner [55] were able to show that in the Miocene of Southern Germany, ruminants with teeth suggesting a browsing diet in a humid wetland environment had less abrasion-dominated mesowear signatures than contemporaneous communities from adjacent drier karst environments. Climate proxy studies by Kaiser & Schulz [16] indicate that this relationship also applies to zebra habitats in sub-Saharan Africa, where plains zebras (*Equus quagga*) from dryer habitats had a more abrasion-dominated mesowear signal than the same species in more humid environments. In contrast, in a study on the influence of different environmental factors on hypsodonty, no influence of climate (wet, mesic or arid) on this trait was found in a sample of 57 mainly African ungulates [15].

Overall, our data do not imply a significant general increase in silica load during the dry season. The fact that even considering grazers only did not lead to a significant relation supports a view of a less than expected effect of changes in rainfall over the seasons on abrasiveness of diets. Other factors, such as grit transport by wind, cover, land erosion and the type of soil will probably have a higher influence on the abrasiveness of plants owing to grit than changes of the seasons.

The occurrence of hypsodonty through time can be regarded as one of the most disputed and fascinating chapters of herbivore evolution. The strong quantitative support of the view of hypsodonty as a signal of ingested silica, and hence abrasiveness, is therefore the major implication and result of this study. While in our data the sum of all silicates was quantified, the elucidation of the contribution of biogenic and external silica to overall intake and the abrasive effect of the respective proportion should be in the focus of future studies.
